# Twelve Weeks of In-Season Strength Training at Moderate Intensities Improve Strength and Body Composition Without Increasing Muscle Damage or Inflammation in Elite Young Female Soccer Players

**DOI:** 10.3390/sports14040136

**Published:** 2026-04-01

**Authors:** Mariem Bousselmi, Amira Ben Moussa Zouita, Manel Darragi, Houssem M. Karamti, Sghaeir Zouita, Juan Del Coso, Ahlem Ben Hmid, Anthony C. Hackney, Urs Granacher, Hassane Zouhal

**Affiliations:** 1 High Institute of Sport and Physical Education of Sfax, University of Sfax, Sfax 3029, Tunisia; b.mariem@yahoo.fr (M.B.); houssemkaramti@hotmail.com (H.M.K.); 2 Research Laboratory (LR23JS01) “Sports Performance, Health & Society”, University of Manouba, Manouba 1003, Tunisia; amira_zouita@yahoo.fr; 3 Higher Institute of Sport and Physical Education of Ksar Said, University of Manouba, Manouba 1003, Tunisia; zouita2003@yahoo.fr; 4 Sport Sciences Research Centre, Rey Juan Carlos University, 28943 Madrid, Spain; juan.delcoso@urjc.es; 5 Laboratory of Transmission, Control and Immunobiology of Infections (LR11IPT02), Pasteur Institute of Tunis, Tunis 1003, Tunisia; a.benhmid@yahoo.com; 6 Faculty of Medicine of Tunis, Tunis El Manar University, Tunis 1003, Tunisia; 7 Department of Exercise & Sport Science, University of North Carolina, Chapel Hill, NC 27599, USA; ach@email.unc.edu; 8 Department of Sport and Sport Science, Exercise and Human Movement Science, University of Freiburg, 79085 Freiburg, Germany; 9 International Institute of Sport Sciences (2I2S), 35000 Rennes, France

**Keywords:** football, exercise training, resistance training, neuromuscular adaptations, blood biomarkers

## Abstract

Strength training (ST) is commonly implemented to enhance soccer-related fitness qualities such as sprinting, jumping, and changes-of-direction while also contributing to injury risk reduction. It is traditionally emphasized in the pre-season period. In-season ST may confer these benefits, but it can also induce muscle damage and inflammation. To examine the effects of a 12-week in-season ST program on maximal dynamic strength, muscle damage biomarkers, and inflammatory biomarkers, 24 elite young female soccer players (Tier 4 according to the McKay et al. classification) aged 14.9 ± 0.8 years and a maturity offset of +2.6 ± 1.1 years were randomly allocated to an ST group (STG, n = 12) or an active control group (CG, n = 12). Both groups followed the same soccer training program. However, in the STG, two weekly soccer sessions were replaced with ST. Overall training volume was comparable between groups. Maximal dynamic strength (1-RM tests for bench press, lat pull-down, and leg press), blood biomarkers of muscle damage (creatine phosphokinase [CPK], lactate dehydrogenase [LDH]), and inflammation (interleukin-6 [IL-6], tumor necrosis factor-α [TNF-α]) were assessed before (T1) and after (T2) the interventions. Analyses showed significant increases for STG for the 1-RM bench press, the 1-RM lat pull-down, and the 1-RM leg press (*p* < 0.001). No significant interactions were detected for any blood marker of muscle damage (LDH and CPK) or inflammation (IL-6 and TNF-α) (all *p* > 0.05). Results support a 12-week in-season ST program improved maximal dynamic strength in elite young female soccer players without altering resting levels of muscle damage or inflammatory markers measured 48 h after training compared to regular soccer training. These findings suggest that ST can be safely implemented during the competitive season in young female soccer players without overreaching or overtraining.

## 1. Introduction

Women’s soccer has experienced a marked increase in global popularity in recent years. According to FIFA’s 2023 report, the number of female soccer players increased by approximately 24% compared to 2019, reaching 16.6 million worldwide [1]. Additionally, the number of professional female players was reported to be 19,064 globally [1]. Modern women’s soccer is now recognized as a high-performance sport requiring a combination of physical conditioning, technical skill, and tactical intelligence. Across all levels of play, soccer involves repeated high-intensity actions, such as sprints, jumps, changes-of-direction, and ball strikes, performed intermittently over ~90 min [2]. For instance, elite female soccer players generally cover between 9,300 and 10,000 m per match, depending on the competition level [3]. Additionally, data from FIFA Women’s World Cups show that the percentage of high-intensity runs (>19 km/h) increased from 16% to 32% between 2015 and 2019, indicating increased physical demands on elite female players [3]. However, while aerobic improvements gained during the pre-season tend to be relatively well-maintained across the competitive season, neuromuscular qualities such as strength and power are more susceptible to declines in the absence of targeted maintenance training [4]. Therefore, key physical attributes, including aerobic endurance, agility, and muscle power, must be systematically developed through targeted training interventions [5,6,7,8]. Among various physical preparation strategies, strength training (ST) has emerged as a key component in women’s soccer due to its role in enhancing high-intensity actions and in reducing injury risk [9]. Additionally, in menstruating female athletes, hormonal fluctuations associated with the menstrual cycle can also represent a significant biological factor influencing training adaptations and performance [10,11]. For example, in terms of training responses, some evidence suggests that delayed-onset muscle soreness and strength loss after exercise are more pronounced during the early follicular phase when estrogen and progesterone levels are low [11,12].

ST is increasingly recognized by coaches and team trainers as a fundamental component of in-season preparation in women’s soccer [7,13]. It has been shown to produce significant benefits in young female players [14]. Incorporating a structured ST program alongside regular soccer practice has been shown to improve several performance attributes in female soccer players. Evidence shows enhancements in muscle strength [5], speed [15], and vertical jump performance [16,17] in youth [16] and adult players [15]. Furthermore, the study by Giustino et al. [18] showed that ST improves performance and prevents injuries in male and female athletes. These adaptations are partly mediated by load- and joint position–dependent changes in movement kinematics, which influence neuromuscular activation and may contribute to both performance enhancement and injury prevention [18].

ST has been shown to induce favorable anthropometric adaptations in elite female athletes, including increases in muscle thickness and muscle fiber cross-sectional area [17]. These hypertrophic muscle responses are supported by repair processes following acute exercise-induced muscle damage, which are partly mediated by inflammatory signaling (e.g., IL-6 and TNF-α) and influenced by training intensity and/or volume [19,20]. In addition to its role in tissue repair, IL-6 acts as a myokine involved in energy metabolism and muscle remodeling during exercise [21,22,23]. Collectively, these physiological responses contribute to neuromuscular adaptations that underpin improvements in strength and power performance [24]. Taken together, the available evidence supports the use of ST as an effective strategy to enhance physical fitness in female soccer players.

In soccer, ST is traditionally emphasized during the pre-season, when training loads are higher, and the focus is toward building physical capacities in preparation for the competitive season [25,26,27]. This phase is typically characterized by relatively high training volumes during the early pre-season, with training intensity progressively increasing as the competitive season approaches to facilitate the development of key physical qualities [26]. However, once the competitive season begins, the emphasis typically shifts toward tactical preparation, match recovery, and injury prevention, often at the expense of maintaining high-intensity ST routines [28]. As a result, the volume and intensity of ST are significantly reduced, challenging any attempt to preserve or further develop muscular strength and power during the season. Coaches prefer to schedule ST only in the pre-season as they are concerned that in-season ST may negatively affect performance through increased muscle damage and inflammation [29,30]. Recent perspectives, such as those presented by Lesinski et al. [7], emphasize that ST should be maintained throughout the competitive season in female youth soccer players to support the development and preservation of strength–power capacities. Furthermore, Darragi et al. [9] demonstrated that in-season ST is an effective method for enhancing physical performance and reducing injury rates among female adolescent soccer players. Few studies have examined the effects of ST on female players’ performance and body composition during the competitive season [7,9,29], and even fewer have examined the effects of ST on biomarkers of inflammation and muscle damage in young female soccer players [29]. Taken together, these findings suggest that the traditional view of limiting ST to the pre-season is evolving, with growing evidence supporting its safe and beneficial application throughout the competitive season.

Consequently, the primary aim of the present study was to determine whether implementing an ST program during the competitive season in elite young female soccer players contributes to performance enhancement or, conversely, whether it may lead to performance deterioration, increased muscle damage, and heightened inflammatory responses. Specifically, the goal was to examine the effects of a 12-week in-season ST program on maximal dynamic strength, muscle damage, and inflammatory markers in elite youth female soccer players. We hypothesized that a well-programmed ST would significantly improve maximal dynamic strength in elite young female soccer players [7,9], without inducing muscle damage or inflammatory responses compared with regular soccer training alone.

## 2. Materials and Methods

### 2.1. Experimental Approach

This study aimed to evaluate the effects of a 12-week ST program, consisting of two weekly ST sessions combined with soccer training during the competitive period, on maximal dynamic strength, as well as the fluctuations in muscle damage and inflammatory markers in elite young female soccer players.

### 2.2. Participants

A minimum total sample size of 20 was determined using G*Power (version 3.1; University of Düsseldorf, Germany), based on an a priori estimate for the primary outcome variable, maximal dynamic strength (one-repetition maximum; 1-RM). The sample size was calculated with a power of 0.9, an alpha level of 0.01, and a moderate effect size of f = 0.49, derived from a related study on 1-RM performance [7]. Twenty-four elite young female soccer players from the Tunisian U15 national team, aged 14.9 years, were recruited and randomly allocated using a simple randomization process to either the strength group (STG; n = 12) or control group (CG; n = 12). Of note, players’ allocation to groups was performed using a computer-generated random sequence by an investigator not involved in testing and training. According to the training and performance caliber framework from McKay et al. [31], the young female soccer players can be classified as Tier 4 (elite). These players were members of the national team that won first place at the North African Union Games in the U15 category, in September 2024. Before participating in the study, none of the players had systematically engaged in ST with overloads. Maturity status was determined using the maturity offset method according to Mirwald et al. [32]. All test- and training-related injuries were systematically monitored and documented by the team’s medical staff throughout the intervention period.

All players followed the standardized training regime prescribed by the national federation staff for the elite U15 national soccer team. The enrolled participants trained for nine months per year, with five weekly training sessions of 90 min each, in addition to one competitive match on the weekend. Concurrently with soccer training, players from both groups attended two one-hour physical education lessons per week as part of their regular school curriculum. During the competitive season, all players trained with the elite U15 national soccer team, but on weekends, they returned to their local academy teams (the clubs from which they had been selected for the national team) to play matches. STG and CG followed the same nutrition and hydration protocols during the soccer season. Dietary and hydration habits were managed by the academy’s nutrition staff, which provided nutritional guidelines for weekend meals. Participants recorded the first day of each menstrual cycle using a calendar-based method to estimate mean cycle duration [10].

All participants were familiarized with the tests implemented in the experimental protocol, and both the players and their parents provided written informed consent before participating in the study. The study was approved by the local ethics committee of the University of Sfax (C.P.P.SUD No. 0494/2023, Tunisia) and by the medical ethics committee of the Tunisian Soccer Federation, in accordance with the latest version of the Declaration of Helsinki. It was also registered in the Pan African Clinical Trials Registry; https://pactr.samrc.ac.za (accessed on 16 January 2026).

### 2.3. Selection Criteria

To be eligible for inclusion, players had to be elite female athletes and members of the U15 national team. Exclusion criteria included the presence of any acute pathology or injury before or during the study period, previous engagement in systematic ST, or receipt of physical therapy or pharmacological treatment. Players with a participation rate of less than 85% in training sessions were excluded. Ultimately, 24 players completed pre- and post-tests.

### 2.4. Experimental Design

**Figure 1:**
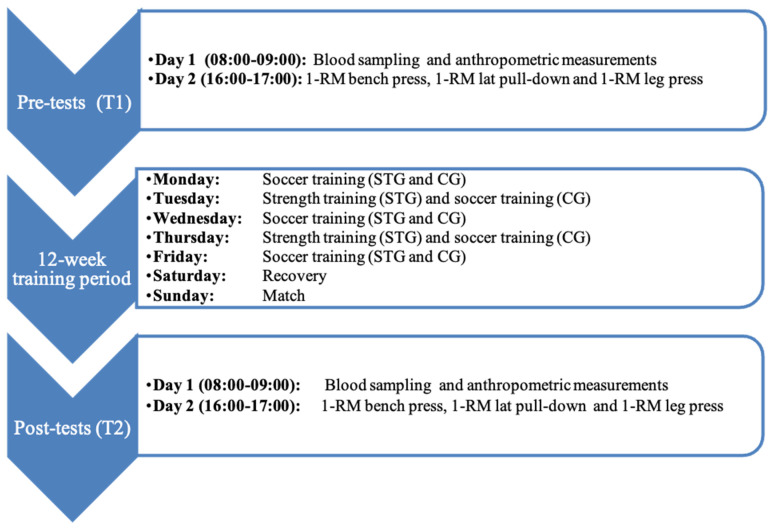
Study Design. T1: before intervention, T2: after intervention; STG: strength training group, CG: control group; 1-RM: one repetition maximum.

This study employed a randomized controlled design with players allocated into either the STG or CG group. The intervention lasted 12 weeks during the competitive season. The STG group performed two weekly ST sessions (each lasting 90 min) in addition to regular soccer practice (i.e., three regular soccer training sessions), with at least 48 h between strength sessions. The CG continued with standard soccer training (i.e., five regular soccer training sessions). To ensure comparable training volumes between groups, overall training load was monitored using session-RPE. Physical assessments, including maximal dynamic strength (1-RM tests), body composition, and blood biomarkers of muscle damage and inflammation, were conducted at baseline and after the intervention to evaluate the physiological and performance effects of in-season ST (see Figure 1).

### 2.5. Procedures

As illustrated in Figure 1, all assessments were conducted at two time points: before (T1) and after (T2) the 12-week intervention. At each time point, assessments were spread over two consecutive days to allow for maximal performance and adequate recovery. On day 1 of each testing phase, blood samples were collected to analyze markers of muscle damage (LDH and CPK) and inflammation (IL-6 and TNF-α), alongside anthropometric and body composition measurements. On day 2, maximal strength tests were performed, including 1-RM measurements for bench press, lat pull-down, and leg press. Prior to each test, participants completed standardized warm-ups and familiarization procedures. Assessors were blinded for all outcome measurements. Anthropometric and body composition measurements were conducted by the medical staff, while maximal strength tests were supervised by strength and conditioning specialists. The experimental design is shown in Figure 1.

### 2.6. Anthropometrics and Body Composition

Body mass was assessed using an impedance-meter scale (Beurer GmbH, BF180, Ulm, Germany, accuracy of ±0.1 kg), and body height was measured using a SECA stadiometer (accuracy ±0.1 cm). Skinfold thickness was measured at four specific locations on the left side of the body (triceps brachii, biceps brachii, subscapular, and suprailiac) to estimate body fat percentage using a Harpenden skinfold caliper (British Indicators Ltd., Luton, UK). Body fat percentage was estimated with the formula outlined by Durnin and Womersley [33]. All measurements were conducted by the same investigator. The ICC for test–retest reliability and the CV for anthropometrics and body composition measured in this study are reported in Table 1.

### 2.7. Maximal Dynamic Strength Tests

Maximal dynamic strength was assessed in the form of the 1-RM before (T1) and after (T2) the 12-week exercise intervention. During the tests, players wore their regular training and competition sports clothing. The 1-RM tests were conducted using weightlifting machines (Matrix, Houdan, France) and included the bench press, lat pull-down, and the leg press. The testing protocol was in accordance with the guidelines of the American College of Sports Medicine (ACSM) [34]. After a general dynamic warm-up of 15 min, participants performed three progressively loaded sets at approximately 40%, 75%, and 85% of their estimated 1-RM, with decreasing repetitions (10-6-3). The first attempt for 1-RM estimation was realized with a load about 5% lower than the estimated value. If successful, an additional load of 5% was used for subsequent attempts until failure occurred after two or three attempts. The highest successful load lifted once would be considered the actual 1-RM. A rest period of 3 min was allowed between attempts and exercises [35]. The ICC for test–retest reliability and the CV for maximal dynamic strength tests measured in this study are provided in Table 2.

### 2.8. Blood Sampling and Biochemical Analyses 

Venous blood samples were collected before (T1) and after (T2) the intervention for both groups. Samples (20 mL) were obtained in the morning (07:00–08:00) following an overnight fast and under controlled ambient conditions (24 ± 1 °C). All participants refrained from intense physical activity for 48 h prior to blood sampling, and post-intervention samples were collected 48 h after the final training session. The blood was drawn into plain tubes (without an anticoagulant) and left to clot at room temperature for 60 min before centrifugation (3000× *g* for 15 min at room temperature). The resulting serum was transferred into Eppendorf tubes and stored immediately at −80 °C until later analysis. Serum CPK and LDH concentrations (IU/L) were measured using the kinetics UV test on a Roche Cobas C system (Cobas Roche^®^), Mannheim, Germany. Serum IL-6 and TNF-α concentrations (pg/mL) were measured using a BD OptEIA Human ELISA (enzyme-linked immunosorbent assay, New York, NY, USA) method. To control for potential reproductive hormonal influences, blood samples were collected during the mid-follicular phase of the menstrual cycle at both T1 and T2 for all participants.

### 2.9. Training Program

ST lasted 12 weeks in-season from December to February and was organized in 3 cycles of four weeks each (Table 3). ST was performed during the first three weeks of each cycle with 2 sessions of ST and 3 sessions of regular soccer training. No ST during the fourth week of the cycle (5 soccer training sessions). Whereas CG practiced their five soccer training sessions per week. Each training session lasted 90 min, with 15 min warm-up and 5 min cool down program. Both groups (STG and CG) followed the same soccer-training program; however, in the STG, two weekly soccer-training sessions were replaced with ST only during the first three weeks of each training cycle. A 48-h rest period was required between the two ST sessions to ensure adequate recovery. ST consisted of six strength exercises (i.e., leg press, hip thrust, squat, bench press, lat pull-down, and calf raises). Participants started with a weight machine to master proper technique before progressing to free weight exercises. Training intensity was progressively increased across the intervention to promote appropriate overload and training progression. During the first training cycle, the intensity was prescribed at 40–60% of the 1-RM based on a strength endurance program emphasizing slow movement execution. STG performed 3 sets of 15 repetitions for each exercise. In the second cycle, intensity was increased to ~60–75% of 1-RM with 3 sets of 12, 10, 8 repetitions for each exercise. The third and final cycle progressed to 85% of 1-RM with 3 sets of 10, 8, 6 repetitions per exercise. The ST program was supervised by a professional strength and conditioning specialist to ensure exercises were performed correctly. The 1-RM was performed every 4 weeks to readjust the load.

### 2.10. Training Load Quantification

The total load of each training session was quantified using the session rating of perceived exertion (s-RPE), which is frequently used for monitoring and tracking athletes’ responses to training loads due to its efficiency, simplicity, and reliability [36]. Players were asked to rate their s-RPE on a 10-point scale, approximately 15 to 20 min following each training session, as previously proposed by Foster and colleagues [36]. Athletes were familiarized with the scale before measurements were taken. A scale with emoticons was also provided to better express the 10 points scale responses. Finally, training load was expressed as -Training load (arbitrary units) = intensity (s-RPE) × session duration (minutes).

### 2.11. Statistical Analyses

The statistical analyses were performed using SPSS software (SPSS, version 22, Chicago, IL, USA) for Windows. To minimize analytical bias, datasets were coded before entry. The statistician performed the initial analyses without knowledge of group allocation. Following completion of the primary analyses, the group codes were revealed, and the results were interpreted according to the group allocation. Results are displayed as means ± standard deviations (SD) for both groups (STG and CG). Data normality was tested using the Shapiro–Wilk test. Non-significant values (*p* > 0.05) were considered indicative of normally distributed data. Homogeneity of variances was assessed using Levene’s test (*p* > 0.05). If data did not follow a normal distribution, logarithmic data transformation was applied [37]. After confirming that the data were normally distributed and that variances were homogeneous, parametric tests were applied to compare the data.

A two-factor analysis of variance (ANOVA) with two groups (STG, CG) and two time points (T1, T2) was used to analyze within and between group comparisons over the twelve-week training period. If a significant group × time interaction effect was detected, Bonferroni-adjusted post hoc tests were computed to indicate group specific pre- to post-changes.

Partial eta squared (η_p_^2^) was used as a measure of effect size and interpreted as small 0.01 ≤ η_p_^2^ < 0.06, medium 0.06 ≤ η_p_^2^ < 0.14, or large ≥ 0.14 [38].

Test–retest reliability was assessed using data from the CG (T1-T2) by means of intraclass correlation coefficients (Cronbach’s ICCs). The ICC values were interpreted as poor (<0.50), moderate (0.50–0.74), good (0.75–0.89), or excellent (≥0.90) test–retest reliability. Statistical significance was set at *p* < 0.05 for all analyses.

## 3. Results

At baseline (T1), no significant differences were observed between the groups (all *p* > 0.05). All participants completed the study protocol, demonstrating adherence rates of 99.2 ± 1.3% in the STG and 98.9 ± 2.1% in the CG. No test- or training-related injuries were reported during the study period.

### 3.1. Training Load

**Figure 2:**
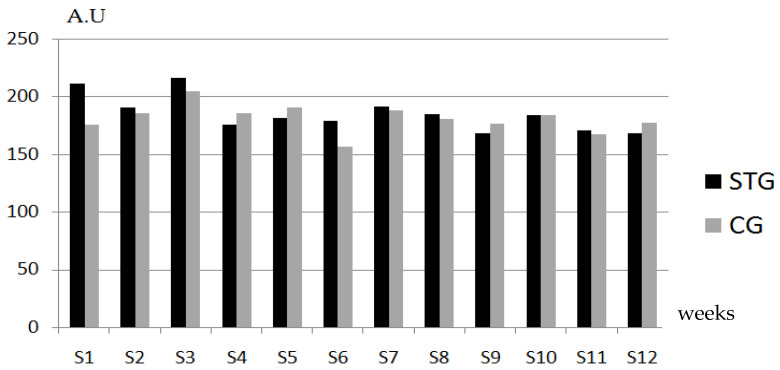
Total training load in elite U15 female soccer players assigned to a strength training or control group; A.U.: arbitrary units, STG: strength training group, CG: control group.

Both groups maintained comparable training loads throughout the 12-week intervention, with the STG accumulating 1168.2 ± 50.8 AU and the CG 1159.1 ± 68.2 AU (*p* = 0.714, n_p_^2^ = 0.006). This equivalence in s-RPE based load confirms matched overall training demands between groups despite differing exercise prescriptions (Figure 2).

### 3.2. Maximal Dynamic Strength

The results of the 1-RM tests are shown in Table 4. There were significant group × time interactions for the 1-RM bench press (*p* = 0.002; n_p_^2^ = 0.37), the 1-RM lat pull-down (*p* < 0.001; n_p_^2^ = 0.60) and the 1-RM leg press (*p* = 0.001; n_p_^2^ = 0.42). Post hoc analyses showed significant increases following ST for the 1-RM bench press (*p* < 0.001; n_p_^2^ = 0.39), the 1-RM lat pull-down (*p* < 0.001; n_p_^2^ = 0.40), and the 1-RM leg press (*p* < 0.001; n_p_^2^ = 0.33) only for STG.

### 3.3. Blood Biomarkers of Muscle Damage and Inflammation

Table 5 displays the effects of 12-week ST on muscle damage and inflammatory biomarkers in elite young female soccer players. No significant group × time interactions were detected for any markers (LDH, CPK, IL-6 or TNF-α; all *p* > 0.05), suggesting similar temporal patterns between groups despite baseline differences in muscle damage markers.

### 3.4. Anthropometrics and Body Composition

After 12 weeks of ST, significant group × time interactions were detected for body mass (*p* = 0.021, n_p_^2^ = 0.22) and percentage body fat (*p* = 0.003, n_p_^2^ = 0.33). Post hoc analyses revealed that the STG experienced significant increases in body mass (*p* < 0.001, n_p_^2^ = 0.02) and reductions in body fat (*p* < 0.001, n_p_^2^ = 0.07), while the CG showed no significant changes in these measures (Table 6).

## 4. Discussion

Our study revealed that twelve weeks of in-season ST led to significant improvements in maximum dynamic strength (1-RM) in elite young female soccer players (Tier 4). Although the observed gains in strength performance are meaningful, they should be interpreted with caution, as part of the training-induced improvements may be attributable to measurement variability and/or learning effects. These results corroborate the findings of the systematic review by Zouita et al. [17], which reported gains of 8 to 19% in maximal strength after 8–12 weeks of ST in elite female athletes. The review of Zouita et al. [17] and previous original studies e.g., [24] noted that improvements were particularly pronounced among ST novices, which helps explain the substantial gains observed in our sample. In line with our findings, Darragi et al. [9] reported increases in maximal strength of 38.9% for 1-RM bench press, 25.6% for lat pull-down, and 55.3% for leg press in young elite female soccer players following 12 weeks of ST, which aligns with the magnitude of change in our current results. Likewise, Fernandez Ortega et al. [39] reported that 12 weeks of pre-season ST (3 times a week with a load equivalent to 80% of 1-RM) significantly improved 1-RM squat performance in young female soccer players. Lastly, the study by Lesinski et al. [7] in elite young female soccer players showed that both power training at moderate to high intensities (50–95% of 1-RM) and strength-endurance training at moderate intensity (50–60% of 1-RM) using machine-based exercises increased 1-RM leg press performance, with greater improvements observed following power training compared to strength-endurance training. Other studies have shown that ST among elite female athletes improves performance in terms of muscle strength, power, and speed variables [40,41]. In addition, the study of Milla et al. [42] showed that adding ST during the season contributes to improved strength in adolescent female soccer players. Furthermore, Lesinski et al. [7] recommend the implementation of ST throughout the soccer season to maintain strength gains in young female soccer players. Although the aforementioned studies reported positive effects of ST on strength gains, this observation is not true for all studies. For example, Grieco et al. [43] observed no significant improvements in maximal isometric strength after a 10-week combined strength, plyometric, and agility training program in female soccer players. These contradictory findings may be due to differences in the applied training methodology, particularly with regard to training intensity, program duration, and exercise selection. In addition, the intervention of Grieco et al. [43] was conducted outside the competitive season and included older players than those in the present study. Such differences in training characteristics may influence the magnitude of neuromuscular adaptations. During the first weeks of training, the observed strength improvements are potentially due to adaptations in the interplay between the central nervous system and the muscle, i.e., improved motor unit recruitment and firing frequency [24]. Taken together, our results highlight the value of introducing ST early in young players’ careers, particularly during the competitive season, to optimize long-term strength gains.

Interestingly, no significant group × time interactions were found for markers of muscle damage or inflammation. This suggests that the addition of ST to soccer training did not cause extra muscular stress compared to regular soccer training only. Our results are consistent with previous research showing minimal fluctuations in these biomarkers during competitive periods. For instance, Peña et al. [44] reported stability in CPK levels among elite adult players throughout a competitive season, and McFadden et al. [30] observed consistent IL-6 and CPK concentrations across different seasonal phases in collegiate athletes. However, Walker et al. [45] reported significant increases in markers of muscle damage and inflammation during the competitive season in female soccer players. These elevations likely reflect the physiological changes imposed by intensive training and insufficient recovery periods during the competitive season [45]. Fluctuations in these biomarkers are suggestive of the physiological changes induced by training [45]. The absence of elevated markers in our study leads us to propose three possibilities for our findings. First, our implemented strength program maintained an optimal load–recovery balance. Secondly, the additional ST did not exacerbate the muscle damage usually observed after intense soccer-specific training and matches. Thirdly, the strength training program was well-tolerated by all players, supporting its safety in youth populations. These results are particularly relevant given that excessive elevations in these biomarkers have been associated with impaired recovery and reduced performance [29]. Taken together, these findings indicate that the implemented in-season ST program was well tolerated and did not impose additional physiological stress beyond that of regular soccer training, supporting its safe integration into the training schedules of elite youth players.

The testing of biomarkers of muscle damage and inflammation provides a tool for coaches and players to detect physiological changes in response to training [45]. Biomarkers of muscle damage and inflammation provide information about the players’ health and performance status and can indicate the current stress and recovery level of players [45]. Monitoring biomarkers of muscle damage and inflammation allows coaches to better control training loads and adjust training to help athletes cope with stress both on and off the pitch. When systematically implemented into training, this means may help improve performance and prevent injuries [29,30,45].

The present study demonstrates that a 12-week in-season ST program also elicited significant improvements in body composition among elite young female soccer players. These robust effect sizes (n_p_^2^ > 0.14) suggest substantial practical significance beyond statistical significance. Our findings corroborate the growing body of evidence supporting ST as an effective modality for body composition optimization in female soccer players [17]. The results are particularly noteworthy given that they were achieved during the competitive season, when training focus typically is toward soccer-specific preparation rather than physical development. The observed adaptations align with previous studies demonstrating similar benefits across different age groups and training protocols. Roso-Moliner et al. [46] reported comparable improvements in adult elite players (24.0 ± 4.2 years) following a 10-week intervention, while Lesinski et al. [7] found positive changes in adolescent players (15 ± 1 years) using strength-endurance protocols. Additionally, the recent study of Darragi et al. [9] on the effects of 12 weeks of ST on body composition in U15 young elite female soccer players demonstrated an increase in overall body mass and lean muscle mass. The consistency of these findings across studies suggests that various ST approaches can effectively modify body composition when properly implemented. However, some studies have failed to demonstrate significant changes [47,48], likely due to differences in training variables including intensity (ranging from 50–85% 1-RM), volume (2–4 sessions/week), exercise selection, or nutritional support. For instance, Campo et al. [47] implemented a 12-week ST program during the latter part of the competitive season, consisting of three training sessions per week, each lasting 40–60 min, in elite female soccer players (22.8 ± 2.1 years), and reported no significant changes in body composition. This lack of differences in body composition can also be explained by the fact that ST focused on lower limb muscles and that female soccer players had previous experience with ST [47]. Authors from other studies did not detect any significant differences after eight weeks of ST on body composition in female soccer players aged 22.1 ± 1.1 years [11]. This disparity in results can be explained by the heterogeneity of body composition among female soccer players at the beginning, maturation, nutrition, different programs, and duration of ST, and genetic endowment [11,17]. Taken together, the present findings suggest that a well-structured in-season ST program can elicit meaningful improvements in body composition in elite youth female soccer players, even when overall training load is matched, and soccer-specific preparation is maintained. The magnitude and consistency of the observed adaptations suggest that the competitive season should not be viewed as a barrier to favorable anthropometric changes when training is appropriately programmed and supervised.

## 5. Conclusions

This study demonstrated that a 12-week in-season ST program using moderate intensities and performed at submaximal velocities can be safely and effectively implemented in elite U15 female soccer players. These findings support that the integration of structured ST during the competitive season can be implemented as a practical strategy to enhance the physical fitness of female youth athletes without inducing any significant changes in the resting levels of muscle damage or inflammatory biomarkers. Importantly, the program was well-tolerated, with high adherence and no reported injuries, underscoring its feasibility in real-world settings. While the results are promising, future research should explore the effects of different ST modalities, assess broader performance outcomes such as sprinting and agility, and investigate long-term impacts on injury prevention and sport-specific performance. Such studies will help refine training prescriptions and potentially optimize athletic development in female soccer across various age groups and competitive levels.

## 6. Limitations

This study does, however, have several limitations that should be acknowledged. First, the sample consisted exclusively of elite female youth soccer players with no prior experience in structured ST. Therefore, the findings may not be generalizable to younger or older age groups, or to athletes with previous exposure to ST. Although all players were classified as post-PHV, individual differences in maturational status may still have influenced training responsiveness and physiological adaptations, potentially affecting the magnitude of the observed outcomes. Second, while the intervention led to improvements in maximal dynamic strength, we did not assess whether these gains translated to enhanced performance in soccer-specific actions such as sprinting, jumping, or changes in direction. Third, the ST protocol employed moderate loads performed at submaximal movement velocity. Consequently, the effects of alternative training modalities, such as low-load high-velocity ST, remain unknown. Moreover, because two soccer-specific sessions were replaced with ST, the experimental group had reduced exposure to technical–tactical and sport-specific stimuli, which may have affected training specificity and limited the ability to determine whether the observed adaptations translate directly to match performance. Fourth, the relatively small sample size and the absence of long-term follow-up restrict the generalizability of the results and prevent conclusions beyond the observed adaptations. In addition, neuromuscular function and direct measures of muscle mass were not assessed, which limits insight into the mechanisms underpinning the physiology of responses to ST and the underlying changes in body composition. Furthermore, internal training load monitoring was based solely on s-RPE. Objective external load metrics (e.g., accelerations and decelerations) and additional internal load indicators such as heart rate–derived measures were not assessed. Finally, some of our inflammatory markers were not assessed at their potential peak physiological responses, which may have compromised the sensitivity of these analyses’ interpretation.

## 7. Practical Applications

From a practical perspective, these findings suggest that carefully prescribed ST can be safely incorporated throughout the competitive season to optimize strength development and reduce injury risk in elite female soccer players. The effectiveness of the program may be attributed to the combination of progressive overload and maintenance of training frequency, which appears to have overcome potential interference effects with the concurrent soccer training. Future research should investigate optimal periodization strategies to maximize these benefits while minimizing interference with technical–tactical development during critical competitive periods.

## Figures and Tables

**Table 1:** Test–retest reliability for anthropometrics and body composition.

Parameters	ICC	95% CI	%CV
Body mass (kg)	0.99	0.97–0.99	3.02%
Body height (cm)	0.99	0.98–0.99	0.43%
BMI (kg.m^−2^)	0.98	0.96–0.99	3.35%
Body fat (%)	0.94	0.87–0.97	4.06%

**ICC:** intra-class correlation, **CI:** confidence interval, **CV:** coefficient of variation, **BMI**: body mass index.

**Table 2:** Test–retest reliability for maximal dynamic strength tests.

Parameters	ICC	95% CI	%CV
1-RM bench press	0.69	0.29–0.86	13.74%
1-RM lat pull-down	0.87	0.72–0.94	19.72%
1-RM leg press	0.87	0.70–0.94	24.00%

ICC: intra-class correlation coefficient, CI: confidence interval, CV: coefficient of variation, 1-RM: one repetition maximum.

**Table 3:** Characteristics of the 12-week in-season strength training program.

	Cycle 1		Cycle 2		Cycle 3	
**Weeks**	**1→3**	**4**	**5→7**	**8**	**9→11**	**12**
Training program	2 strength training sessions + 3 soccer training sessions	5 soccer training sessions	2 strength training sessions + 3 soccer training sessions	5 soccer training sessions	2 strength training sessions + 3 soccer training sessions	5 soccer training sessions
% RM	**40→60%**	recovery	**60→75%**	recovery	**→85%**	recovery
Number of set/ Number of repetitions	3 sets 15 repetitions		3 sets 12-10-8		3 sets 10-8-6	

%RM: % of one- repetition maximum (training intensity); Cycle: 4 weeks of training; recovery: no strength training, only soccer training.

**Table 4:** Maximal dynamic strength tests of elite U15 female soccer players following a 12-week in-season intervention.

Variables	Group	T1	T2	*p* Value (Partial η^2^)		
				Time Effect	Group Effect	Interaction Effect
Bench press (kg)	STG	35.7 ± 4.4	44.1 ± 5.8	**<0.001** (0.64)	0.068 (0.14)	**0.002** (0.37)
	CG	35.2 ± 4.6	37.5 ± 4.7			
Lat pull-down (kg)	STG	21.9 ± 3.9	30.1 ± 5.8	**<0.001** (0.87)	**0.005** (0.30)	**<0.001** (0.61)
	CG	19.1 ± 3.9	22.0 ± 3.7			
Leg press (kg)	STG	63.2 ± 20.9	98.4 ± 28.0	**<0.001** (0.84)	**0.008** (0.27)	**0.001** (0.42)
	CG	51.1 ± 11.3	67.1 ± 11.2			

Data are presented as means and standard deviation (±SD), T1: before intervention, T2: after intervention, STG: strength training group, CG: control group, bold values: *p* < 0.05. Results are shown for the bench press, lat pull-down, and leg press in the strength training group (STG, n = 12) and the control group (CG, n = 12).

**Table 5:** Muscle damage and inflammatory biomarkers of elite U15 female soccer players following a 12-week in-season intervention.

Variables	Group	T1	T2	*p* Value (Partial η^2^)		
				Time Effect	Group Effect	Interaction Effect
LDH (IU/L)	STG	186.8 ± 22.1	193.2 ± 25.5	0.835 (0.00)	0.019 (0.22)	0.310 (0.04)
	CG	173.4 ± 24.0	169.2 ± 14.6			
CPK (IU/L)	STG	172.8 ± 63.8	235.5 ± 92.7	0.016 (0.23)	0.009 (0.27)	0.361 (0.03)
	CG	117.4 ± 54.2	147.2 ± 82.7			
IL-6 (pg/mL)	STG	16.1 ± 6.2	15.1 ± 4.4	0.243 (0.07)	0.051 (0.18)	0.107 (0.13)
	CG	10.0 ± 2.9	15.1 ± 5.2			
TNF-α (pg/mL)	STG	12.8 ± 3.9	13.2 ± 6.0	0.749 (0.00)	0.593 (0.01)	0.350 (0.04)
	CG	12.9 ± 4.8	13.3 ± 5.9			

Data are presented as means and standard deviation (±SD), T1: before intervention, T2: after intervention, LDH: Lactate dehydrogenase, CPK: Creatine phosphokinase, IL-6: interleukin 6, TNF-α: Tumor necrosis factor alpha, STG: strength training group, CG: control group. Values are presented for the strength training group (STG, n = 12) and the control group (CG, n = 12).

**Table 6:** Anthropometric changes in elite U15 female soccer players following a 12-week in-season strength training intervention.

Variables	Group	T1	T2	*p* Value (Partial η^2^)		
				Time Effect	Group Effect	Interaction Effect
Body mass (kg)	STG	55.2 ± 8.9	57.8 ± 9.4	<0.001 (0.64)	0.756 (0.00)	0.021 (0.22)
	CG	57.0 ± 7.5	58.2 ± 7.3			
Body height (cm)	STG	163.5 ± 7.6	163.9 ± 7.6	0.916 (0.00)	0.926 (0.00)	0.054 (0.15)
	CG	163.6 ± 6.2	163.3 ± 6.7			
BMI (kg/m^2^)	STG	20.5 ± 3.1	21.5 ± 2.7	<0.001 (0.61)	0.637 (0.01)	0.058 (0.15)
	CG	21.3 ± 2.4	21.8 ± 2.5			
Body fat (%)	STG	26.0 ± 2.9	24.4 ± 2.9	<0.001 (0.44)	0.211 (0.07)	0.003 (0.33)
	**CG**	26.7 ± 2.5	26.5 ± 2.0			

Data are presented as means and standard deviation (±SD), T1: before intervention, T2: after intervention, BMI: Body mass index, STG: strength training group, CG: control group. Values are presented for the strength training group (STG, n = 12) and the control group (CG, n = 12).

## Data Availability

The datasets generated and analyzed during the current study are not publicly available due to confidential information about the participants, but are available from the corresponding author on reasonable request.

## Abbreviations

The following abbreviations are used in this manuscript:

ST	Strength training
STG	Strength training group
CG	Active control group
1-RM	One-repetition maximum
T1	Before intervention,
T2	After intervention
BMI	Body mass index
ACSM	American College of Sports Medicine
CV	Coefficients of variation
CI	Confidence interval
AU	Arbitrary units
ANOVA	Analysis of variance
ICC	Intraclass correlation coefficient
SD	Standard deviations
s-RPE	Session-rating of perceived exertion
CPK	Creatine phosphokinase
LDH	Lactate dehydrogenase
IL-6	Interleukin-6
TNF-α	Tumor necrosis factor-α

## References

[1] FIFA (2023). Women’s Football: Member Association Survey Report 2023. https://inside.fifa.com/womens-football/member-associations-survey-report-2023.

[2] Vescovi J.D., Fernandes E., Klas A. (2021). Physical demands of women’s soccer matches: A perspective across the developmental spectrum. Front. Sports Act. Living.

[3] Gabbett T.J., Mulvey M.J. (2008). Time-motion analysis of small-sided training games and competition in elite women soccer players. J. Strength Cond. Res..

[4] Belamjahad A., Tourny C., Jebabli N., Clark C.C.T., Laher I., Hackney A.C., Granacher U., Zouhal H. (2024). Effects of a preseason neuromuscular training program vs. An endurance-dominated program on physical fitness and injury prevention in female soccer players. Sports Med. Open.

[5] Bergmann F., Gray R., Wachsmuth S., Höner O. (2021). Perceptual-Motor and Perceptual-Cognitive Skill Acquisition in Soccer: A Systematic Review on the Influence of Practice Design and Coaching Behavior. Front. Psychol..

[6] Filipas L., Borghi S., La Torre A., Smith M.R. (2021). Effects of mental fatigue on soccer-specific performance in young players. Sci. Med. Footb..

[7] Lesinski M., Prieske O., Chaabene H., Granacher U. (2021). Seasonal Effects of Strength Endurance vs. Power Training in Young Female Soccer Athletes. J. Strength Cond. Res..

[8] Williams R.A., Cooper S.B., Dring K.J., Hatch L., Morris J.G., Sunderland C., Nevill M.E. (2020). Effect of football activity and physical fitness on information processing, inhibitory control and working memory in adolescents. BMC Public Health.

[9] Darragi M., Zouhal H., Bousselmi M., Karamti H.M., Clark C.C., Laher I., Hackney A.C., Granacher U., Zouita A.B. (2024). Effects of In-Season Strength Training on Physical Fitness and Injury Prevention in North African Elite Young Female Soccer Players. Sports Med. Open.

[10] Igonin P.H., Rogowski I., Boisseau N., Martin C. (2022). Impact of the menstrual cycle phases on the movement patterns of sub-elite women soccer players during competitive matches. Int. J. Environ. Res. Public Health.

[11] Randell R.K., Clifford T., Drust B., Moss S.L., Unnithan V.B., De Ste Croix M.B.A., Datson N., Martin D., Mayho H., Carter J.M. (2021). Physiological characteristics of female soccer players and health and performance considerations: A narrative review. Sports Med..

[12] Romero-Parra N., Cupeiro R., Alfaro-Magallanes V.M., Rael B., Rubio-Arias J.Á., Peinado A.B., Benito P.J., on behalf of the IronFEMME Study Group (2021). Exercise-induced muscle damage during the menstrual cycle: A systematic review and meta-analysis: A systematic review and meta-analysis. J. Strength Cond. Res..

[13] Lesinski M., Prieske O., Helm N., Granacher U. (2017). Effects of Soccer Training on Anthropometry, Body Composition, and Physical Fitness during a Soccer Season in Female Elite Young Athletes: A Prospective Cohort Study. Front. Physiol..

[14] Moran J., Sandercock G., Ramirez-Campillo R., Clark C.C., Fernandes J.F., Drury B. (2018). A Meta-Analysis of Resistance Training in Female Youth: Its Effect on Muscular Strength, and Shortcomings in the Literature. Sports Med..

[15] Vikmoen O., Rønnestad B.R., Ellefsen S., Raastad T. (2017). Heavy strength training improves running and cycling performance following prolonged submaximal work in well-trained female athletes. Physiol. Rep..

[16] Lesinski M., Prieske O., Granacher U. (2016). Effects and dose–response relationships of resistance training on physical performance in youth athletes: A systematic review and meta-analysis. Br. J. Sports Med..

[17] Zouita A., Darragi M., Bousselmi M., Sghaeir Z., Clark C.C., Hackney A.C., Granacher U., Zouhal H. (2023). The Effects of Resistance Training on Muscular Fitness, Muscle Morphology, and Body Composition in Elite Female Athletes: A Systematic Review. Sports Med..

[18] Giustino V., Vicari D.S., Figlioli F., Gervasi M., Fernández Peña E., Schifaudo N., Tedesco M., Drid P., Paoli A., Battaglia G. (2024). Kinematic analysis of the back squat at different load intensities in powerlifters and weightlifters. Front. Sports Act. Living.

[19] Suzuki K., Tominaga T., Ruhee R.T., Ma S. (2020). Characterization and modulation of systemic inflammatory response to exhaustive exercise in relation to oxidative stress. Antioxidants.

[20] Owens D.J., Twist C., Cobley J.N., Howatson G., Close G.L. (2019). Exercise-induced muscle damage: What is it, what causes it and what are the nutritional solutions?. Eur. J. Sport. Sci..

[21] Kistner T.M., Pedersen B.K., Lieberman D.E. (2022). Interleukin 6 as anenergyallocator in muscletissue. Nat. Metab..

[22] Orange S.T., Leslie J., Ross M., Mann D.A., Wackerhage H. (2023). The exercise IL-6 enigma in cancer. Trends Endocrinol. Metab..

[23] Zunner B.E., Wachsmuth N.B., Eckstein M.L., Scherl L., Schierbauer J.R., Haupt S., Stumpf C., Reusch L., Moser O. (2022). Myokines and resistance training: A narrative review. Int. J. Mol. Sci..

[24] Mason J., Frazer A.K., Avela J., Pearce A.J., Howatson G., Kidgell D.J. (2020). Tracking the corticospinal responses to strength training. Eur. J. Appl. Physiol..

[25] Loturco I., Pereira L.A., Reis V.P., Zanetti V., Bishop C., McGuigan M.R. (2022). Traditional Free-Weight vs. Variable Resistance Training Applied to Elite Young Soccer Players During a Short Preseason: Effects on Strength, Speed, and Power Performance. J. Strength Cond. Res..

[26] Thomakos P., Spyrou K., Katsikas C., Geladas N.D., Bogdanis G.C. (2023). Effects of Concurrent High-Intensity and Strength Training on Muscle Power and Aerobic Performance in Young Soccer Players during the Pre-Season. Sports.

[27] Wong P., Chaouachi A., Chamari K., Dellal A., Wisloff U. (2010). Effect of Preseason Concurrent Muscular Strength and High-Intensity Interval Training in Professional Soccer Players. J. Strength Cond. Res..

[28] Loturco I., Freitas T.T., Alcaraz P.E., Kobal R., Nunes R.F., Weldon A., Pereira L.A. (2021). Practices of strength and conditioning coaches in Brazilian elite soccer. Biol. Sport..

[29] McFadden B.A., Walker A.J., Arent M.A., Bozzini B.N., Sanders D.J., Cintineo H.P., Bello M.L., Arent S.M. (2020). Biomarkers Correlate with Body Composition and Performance Changes Throughout the Season in Women’s Division I Collegiate Soccer Players. Front. Sports Act. Living.

[30] McFadden B.A., Walker A.J., Bozzini B.N., Hofacker M., Russell M., Arent S.M. (2022). Psychological and Physiological Changes in Response to the Cumulative Demands of a Women’s Division I Collegiate Soccer Season. J. Strength Cond. Res..

[31] McKay A.K., Stellingwerff T., Smith E.S., Martin D.T., Mujika I., Goosey-Tolfrey V.L., Sheppard J., Burke L.M. (2021). Defining training and performance caliber: A participant classification framework. Int. J. Sports Physiol. Perform..

[32] Mirwald R.L., Baxter-Jones A.D., Bailey D.A., Beunen G.P. (2002). An assessment of maturity from anthropometric measurements. Med. Sci. Sports Exerc..

[33] Durnin J.V., Womersley J. (1974). Body fat assessed from total body density and its estimation from skinfold thickness: Measurements on 481 men and women aged from 16 to 72 Years. Br. J. Nutr..

[34] Ferguson B. (2014). ACSM’s Guidelines for Exercise Testing and Prescription 9th Ed. 2014. J. Can. Chiropr. Assoc..

[35] Lockie R.G., Dawes J.J., Jones M.T. (2018). Relations between linear speed and lower-body power with change-of-direction speed in national collegiate athletic association divisions I and II women soccer athletes. Sports.

[36] Foster C., Boullosa D., McGuigan M., Fusco A., Cortis C., Arney B.E., Orton B., Dodge C., Jaime S., Radtke K. (2021). 25 Years of Session Rating of Perceived Exertion: Historical Perspective and Development. Int. J. Sport. Perform..

[37] Feng C., Wang H., Lu N., Chen T., He H., Lu Y., Tu X.M. (2014). Log-transformation and its implications for data analysis. Shanghai Arch. Psychiatry.

[38] Hopkins W.G., Marshall S.W., Quarrie K.L., Hume P.A. (2007). Risk factors and risk statistics for sports injuries. Clin. J. Sport. Me.

[39] Ortega J.A.F., De los Reyes Y.G., Peña F.R.G. (2020). Effects of strength training based on velocity versus traditional training on muscle mass, neuromuscular activation, and indicators of maximal power and strength in girls soccer players. Apunt. Sports Med..

[40] Chamera T., Prończuk M., Smok P., Drozd M., Michalczyk M., Maszczyk A. (2023). The effects of resistance training on jumping and selected power variables of the lower limbs in female soccer players. Balt. J. Health Phys. Act..

[41] Ruivo R.M., Carita A.I., Pezarat-Correia P. (2016). Effects of a 16-week strength-training program on soccer players. Sci. Sports.

[42] Millar N.A., Colenso-Semple L.M., Lockie R.G., Marttinen R.H.J., Galpin A.J. (2020). In-season hip thrust vs. back squat training in female high school soccerplayers. Int. J. Exerc. Sci..

[43] Grieco C.R., Cortes N., Greska E.K., Lucci S., Onate J.A. (2012). Effects of a combined resistance-plyometric training program on muscular strength, running economy, and V[Combining Dot Above]O2peak in division I female soccer players. J. Strength Cond. Res..

[44] Peña N., Amézaga J., Marrugat G., Landaluce A., Viar T., Arce J., Larruskain J., Lekue J., Ferreri C., Ordovás J.M. (2023). Competitive season effects on polyunsaturated fatty acid content in erythrocyte membranes of female football players. J. Int. Soc. Sports Nutr..

[45] Walker A.J., McFadden B.A., Sanders D.J., Rabideau M.M., Hofacker M.L., Arent S.M. (2019). Biomarker response to a competitive season in division I female soccer players. J. Strength Cond. Res..

[46] Roso-Moliner A., Mainer-Pardos E., Arjol-Serrano J.L., Cartón-Llorente A., Nobari H., Lozano D. (2022). Evaluation of 10-week neuromuscular training program on body composition of elite female soccer players. Biology.

[47] Campo S.S., Vaeyens R., Philippaerts R.M., Redondo J.C., de Benito A.M., Cuadrado G. (2009). Effects of Lower-Limb Plyometric Training on Body Composition, Explosive Strength, and Kicking Speed in Female Soccer Players. J. Strength Cond. Res..

[48] Dutta M., Rawat J., Dwivedi V. (2018). Effect of eight weeks strength training on various variables of body composition of female football players. Indian J. Phys. Educ. Sports Med. Exerc. Sci..

